# Dr. Charlesworth and Mr. Gardiner Hill; or the Non-Restraint System of Treatment in Lunacy

**Published:** 1854-01-01

**Authors:** 


### DR. CHARLESWORTH AND MR. GARDINER HILL; OR THE NON-RESTRAINT SYSTEM OF TREATMENT IN LUNACY.

It is of vital importance to the best interests of society that questions of priority in the discovery of great truths should be decided with impartiality and justice. A question of priority has been lately contested with a warmth not very unusual in matters of this kind. Mr. Gardiner Hill (formerly house surgeon to the Lincoln Asylum, under the late Dr. Charlesworth, the visiting physician to that institution) industriously advertises himself as the "*originator of the non-restraint system*" in the treatment of insanity. Mr. Hill, as we understand his position, prefers this claim to the extent of virtually excluding all share in this "discovery" to Dr. Charlesworth. How does the matter stand? In the first place, can we call the "Non-Restraint" system, as expounded by Mr. Hill, a "discovery"? It may fairly be questioned how far a system of treatment which consists to some extent in the substitution of manual force for instrumental coercion, is entitled to this high-sounding appellation. In the second place, is it enough to have *enunciated*, in an anticipative sense, the proposition, "that it may be possible to conduct an institution for the insane without having recourse to any instruments of restraint whatever,"—is it enough, we say, to hazard this conjecture, in order to establish a claim to a "discovery" of a principle hitherto unknown? In the third place, did Mr. Hill stamp this presumed discovery with the character of a lasting truth, by carrying his anticipative speculation into successful practice? We are compelled to answer that he failed to accomplish this upon his own showing—and we cannot admit that any explanation of necessity, however plausible, can in any way destroy the fact, or weaken the inference from it.

That Mr. Hill generally evinced, throughout his official career, an earnest endeavour to extend the application of the humane principle of treatment for which the Lincoln Asylum had for many years previously to his time been renowned, we willingly admit. That the authorities, and no one more heartily and unreservedly than Dr. Charlesworth himself, repeatedly recorded their approbation of his zeal, is also true. It would be unjust to deny to Mr. Hill this merit. But that he made any "discovery" in the matter is most absurd. That he initiated, either in theory or in practice, the system of non-restraint, is also opposed to historic evidence. To assert that Dr. Charlesworth ever transferred from himself to Mr. Hill the honour of this said "discovery," is also untrue. None of the expressions of Dr. Charlesworth, so much relied upon by Mr. Hill, will bear this forced construction. It is, on the contrary, within our own knowledge, that Dr. Charlesworth, who was ever ready, in the most generous manner, to extol Mr. Hill's merits, expressly and emphatically denied that he was entitled to the particular honour which he now claims. We also know that those physicians whose labours in this department of science entitle their opinions to respect, entertain the same view upon the subject. In justice to the honoured name of Dr. Charlesworth, we cannot help expressing our conviction that these gentlemen are bound to speak out before the public that which they have not hesitated to say in private.*

But apart entirely from these considerations we ask, in sober seriousness, whether on other grounds Mr. Hill has any right to put himself forward as the "discoverer," or "originator" of the "non-restraint system of treating the insane"? The great "discoverer," the illustrious "originator," was unquestionably the immortal PINEL; but this FACT appears to be entirely ignored and lost sight of by those who have busied themselves in this noisy controversy. Mr. Gardiner Hill may have *adopted and carried out in this country a principle of treatment* developed by this celebrated physician;† but he has no more claim to the designation of "originator" or "discoverer," *quoad* the abolition of restraint, than a man has to call himself the "originator" of vaccination, simply because he enforces the importance of Jenner's discovery, as a protection against small-pox! Does not Mr. Hill expose himself to the charge of arrogance and presumption by designating himself, as he ostentatiously and absurdly does in all his advertisements, as the *author* (?) *and originator of the non-restraint system of treatment in lunacy.*"‡ We never read his often-repeated advertisements without a feeling of pain and humiliation. Let the honoured mantle fall upon the right shoulders; let the revered name of Pinel have all the credit of the "discovery" or "authorship," as Mr. Hill ridiculously terms it; but, for God's sake, let it not be said that any *Englishman* endeavoured by stealth to filch from the immortal Frenchman the honour to which he is so justly entitled, and which the whole civilized world awards to him, for first recognising the important principle that it is possible in the treatment of the insane to dispense (in a great measure) with the use of mechanical restraint! If Mr. Hill asserts that Pinel only took the *initiative*—the first step in the matter, and that it remained for him (Mr. Hill) to *mature* the discovery, and to establish that it is possible to treat *all* cases of insanity without mechanical restraint, then we understand his position and can appreciate the validity of his claim to the title of "originator," "author," and "discoverer." If we are right in our estimate of Mr. Hill's much-

* The question has further been put in the clearest light in an able and faithful summary of the history of the Lincoln asylum in "The Lancet." The conclusion from that summary is irresistible.

† It is a curious fact, and one not generally known, that the idea of carrying out a more humane system of treating the insane originated with two men whose names are seldom mentioned in connexion with this subject. We refer to *Tenon* and *Rouchefoucald.* But this fact does not detract from the great credit due to *Pinel* for his heroic exertions at the Bicêtre, where the experiment was first tried. The subjoined passage refers to the fact:—"Enfin, en 1768. . . . Tenon publia un mémoire rémarquable, dans lequel se trouvent indiquées les premièrees notions d'un régime doux et humain pour les aliénés. Un citoyen, La Rouchefoucald, en comprit toute la portée, et joignit plus tard ses efforts aux siens, en faisant en 1791, à l'assemblée constituante, plusieurs rapports, qui dévoilaient l'état misérabl dans lequel languissaient les aliénés. C'était là sans doute la douleur d'une belle âme et de nobles efforts auxquels il est juste de renvoyer la cause première des amélioprations que Pinel put l'executer l'année suivante à Bicêtre."—Scipion Pinel, "*Traité Complet du Régime Sanitaire des Aliénés.*" 1836. Pp. 55, 56.

‡ *Vide* the Medical Directories.

vaunted "discovery," then we will only now say, that if it be his deliberately formed opinion that no case of acute insanity can possibly occur in which the application of restraint would be justifiable—if such be his dictum, he will find himself opposed to the united experience of all the practical physicians of England, France, Germany, and America.

We have ourselves heard Dr. Conolly (who has always been deemed in this country the leading advocate of the "non-restraint" system of treating the insane) declare, in a court of law, that "mechanical restraint" could not invariably be dispensed with; that cases will present themselves in which it may be necessary to have recourse to it.* We may, in a few words, take our leave of this disagreeable subject. We have stated that all the honour of this "discovery" is, beyond all question, due to Pinel. Nor can any one of reflection and experience admit the possibility of one man having conceived the splendid project of working such a mighty revolution against the tyranny of opinion and the inertia of custom, and of carrying out this work to practical perfection in the course of a few years. More than half a century has expired since the first step was taken by Pinel: and the gigantic labour has not yet reached its consummation. Many minds have been earnestly striving to forward the good work. Foremost amongst these, in this country, must ever stand the name of Charlesworth. For thirty-five years he never wearied in the task. If to the Lincoln system is due the high honour of having shown a bright example of what may be done in the abolition of barbarous instruments, that honour it owes to Dr. Charlesworth. To render this abolition feasible, how many reforms were necessary! The whole physical and moral aspect of the scene had to be changed. This was a work of time—of devoted patience—of never-failing courage and perseverance. This was the work of Dr. Charlesworth. Let the applause of his fellow-men—the only reward he can now have—be accorded to him.

If ever the motto of Lord Somers, "*Prodesse quàm conspici*," could be justly assumed by any other man, that man was the late Dr. Charlesworth. We hope it may not be interpreted as evidence of an unkind feeling towards Mr. Hill—a feeling we altogether disclaim—if we invite his attention to this admirable maxim. It is doubtful whether any man ever made good a claim to priority by blazoning his pretensions before the world in the shape of advertisements. Such a course of proceeding will hardly promote his cause amongst men of science and reflection, and is not altogether free from injurious imputations.

*If* Mr. Hill be the "author and originator of the non-restraint system of treatment in lunacy"—*if* he really did make this great discovery—then we ask, is it professional or even decent for him to parade this fact in the advertisements that announce his being the proprietor of a private asylum? If he be the "coming man," the psychological star "looming in the future," the world will not be long in recognising his merits, and thus release this gentleman from the fatigue and inconvenience necessarily consequent upon his being always obliged to blow his own trumpet!

* The trial of "Hill *v.* Philp," Court of Queen's Bench.

